# Cytotoxic immune cells do not affect TDP-43 and p62 sarcoplasmic aggregation but influence TDP-43 localisation

**DOI:** 10.1038/s41598-023-42824-5

**Published:** 2023-09-23

**Authors:** Bryony McCord, Richard M. Day

**Affiliations:** Centre for Precision Healthcare, UCL Division of Medicine, University College London, London, WC1E 6JF UK

**Keywords:** Cell biology, Immunology, Diseases

## Abstract

Sporadic inclusion body myositis (sIBM) is an idiopathic inflammatory myopathy with invasion of CD8 T cells in muscle and aggregation of proteins in the sarcoplasm. TDP-43 and p62 are two proteins that aggregate in affected muscle, and have been suggested as specific markers for sIBM over other inflammatory myopathies. TDP-43 is also mislocalised from the nucleus to the sarcoplasm in sIBM. It is not clear if inflammation precedes protein aggregation in sIBM. This study investigated if exposure to cytotoxic inflammatory cells caused TDP-43 and p62 aggregation or TDP-43 mislocalisation in cultured myotubes. TALL-104 coculture was highly cytotoxic to myotubes after 24 h. Secretion of IFNγ and TNFα were higher in cocultures compared to monocultured TALL-104 cells, indicating activation. TALL-104 cells attached to and infiltrated myotubes. There was no effect of TALL-104 coculture on TDP-43 or p62 sarcoplasmic aggregate size or frequency. However, there was decreased localisation of TDP-43 to the nucleus with TALL-104 coculture compared to control. In an in vitro setting, cytotoxic immune cells did not cause TDP-43 or p62 sarcoplasmic aggregation, suggesting cellular cytotoxicity may not trigger aggregation of these proteins. However TALL-104 coculture influenced TDP-43 localisation, suggesting cytotoxic immune cells may contribute to TDP-43 localisation shifts which is observed in sIBM.

Sporadic inclusion body myositis (sIBM) is the most common form of myositis in people over the age of 50^1,2^, yet is a rare disease with a prevalence of 3.5–18.2 in those over 50 years^3–5^. The disease is characterised by muscle weakness especially in the quadriceps and finger flexors, with presentation usually asymmetrical affecting the non-dominant limb more^6^. It is slowly progressive and many patients requiring mobility aids at later stages, with the average time to requiring mobility aids of 10 years for a walking stick^7^ and 10–15 years for a wheelchair^5,7^. A common progressive symptom is dysphagia, which occurs in up to 40–86% of cases and can be a cause for mortality^7–9^.

sIBM remains an idiopathic disease with no singular distinct cause and many mechanisms implicated in this disease. Broadly, pathological features of sIBM can be split into inflammatory and non-inflammatory/degenerative. Within affected skeletal muscle fibres of sIBM patients there is infiltration of inflammatory cells, mostly CD8 + cytotoxic T cells, into the endomysium and the muscle fibres themselves^2,10^. These CD8 + T cells are minimally proliferative and differentiated towards an effector cell phenotype with high cytotoxicity^11^. The inhibitory receptor and marker of differentiated CD8 cells, killer cell lectin-like receptor G1 (KLRG1), is highly expressed on muscle-invading and circulating CD8 T cells in sIBM patients, and is co-expressed with cytotoxic genes^11^. Other T cell differentiation markers have been identified in sIBM cytotoxic T cells, including the senescent-associated CD57^1,12,13^ and the differentiation marker T-bet^1^. As well as inflammation, sIBM is also characterised by degenerative non-inflammatory features. This includes the presence of rimmed vacuoles in affected fibres^14^, inclusion bodies and protein aggregates within the sarcoplasm and rimmed vacuoles^15^. Two proteins that are aggregated and potentially dysregulated in sIBM muscle are TDP-43 and p62.

TDP-43 is an RNA/DNA binding protein involved in mRNA transport, stability, splicing regulation^16^, and stress sensing^17^. It is also involved in the removal of cryptic exons from some mRNAs^18^. Cryptic exons are usually excluded from mature mRNAs molecules^19^, and their inclusion in mature mRNAs can lead to splice variants causing premature stop codons or frameshift mutations.

TDP-43 is aggregated in the sarcoplasm in sIBM^20,21^. TDP-43 was found accumulated in all biopsies of definite sIBM cases and 31% of possible sIBM cases^20^, whilst a separate study found 67% of sIBM cases were positive for TDP-43 aggregates^22^. sIBM patients also show higher levels of 25 and 35 kDa truncated fragments of TDP-43 compared to healthy controls^23^. The nuclear localisation of TDP-43 is decreased in sIBM compared to healthy controls^21^. The presence of TDP-43 within the sarcoplasm with a lack of nuclear TDP-43 has been suggested as a marker of sIBM, with its presence detected in 25% of sIBM myofibres, whereas rimmed vacuoles were detected in 2.8% of sIBM myofibres^22,24^. It is not clear how TDP-43 aggregation and cytoplasmic mislocalisation may contribute to sIBM pathology. Potentially, cytosolic sequestration of TDP-43 in aggregates causes TDP-43 to lose its homeostatic functions. Indeed, sIBM patient muscle has widespread alterations in RNA metabolism pathways compared to controls^25^. sIBM muscle has altered TDP-43-controlled cryptic exon inclusions compared to control patients in ASAP2 (ArfGAP with SH3 domain, ANK repeat and PH domain 2-containing protein 2)^26^. Furthermore, detection of cryptic exons in sIBM muscle was 84% sensitive and 99% specific for sIBM compared to healthy control and other muscle disease patients^27^.

p62 is involved in selective autophagy and the ubiquitin proteasome system (UPS)^28^. As with TDP-43, p62 is aggregated within the sarcoplasm of sIBM patients^10,20^, and p62 aggregates are often associated with vacuolated areas in affected sIBM fibres^29,30^. Myofibres with high p62 levels also show high levels of TDP-43 and mitochondrial staining^23^. p62 aggregates in sIBM muscle can be large, and approximately half of the p62 aggregates colocalised with LC3, suggesting a potential role for defective selective autophagy in sIBM^31^. The combined presence of TDP-43 and p62 in sIBM muscle has been suggested as a specific marker for sIBM over other inflammatory myopathies^20–22^.

Currently it is not known whether degenerative or inflammatory features of sIBM arise first, and if there is an interaction between these features. Knowing if inflammation or protein aggregation precedes other characteristics would help the understanding of this disease and could help in the development of treatments targeted towards the initiating events in sIBM pathology. Here, coculture of myotubes and the human cytotoxic cell line TALL-104 was used, which triggers cytotoxicity via a major histocompatibility complex (MHC)-independent pathway through perforin, granzyme, Fas ligand, and TRAIL (TNF-related apoptosis-inducing ligand)^32,33^. This study aims to investigate in an in vitro system whether exposure to cytotoxic immune cells in the form of the TALL-104 can trigger non-inflammatory features observed in sIBM, focussing on TDP-43 and p62 sarcoplasmic aggregation and TDP-43 mislocalisation.

## Methods

All materials were purchased from ThermoFisher Scientific, UK unless otherwise stated.

### Cell culture

Skeletal muscle-derived cells from Lonza Clonetics™ (UK, CC-2580) or Cook MyoSite® (USA, SK-1111) from healthy donors were grown in Ham’s F10 nutrient mix (11550043) supplemented with 20% FBS (10500064), 1 µM dexamethasone (Sigma, UK), 10 ng/mL basic fibroblast growth factor (bFGF, Peprotech, UK 100-18B), and 1% penicillin–streptomycin. Skeletal muscle-derived cells were differentiated in N2 differentiation medium: DMEM/F12 1:1 (11320074), 1% N2 supplement (17502048), 1% L-glutamine, 1% insulin-transferrin-selenium (41400045), and 0.2% penicillin–streptomycin. TALL-104 cells were purchased from LGC standards, UK. TALL-104 cells were cultured in ATCC-modified RPMI with 20% FBS and 20 ng/mL IL-2 premium grade (Milltenyi Biotec, UK 130–097-744).

Myotubes were seeded at either 4 × 10^4^ cells per 24-well with two days proliferation or 5.5 × 10^4^ cells per well with one day proliferation followed by 5 days differentiation. TALL-104 cells were centrifuged at 170×* g* and resuspended in N2 myotube differentiation medium with 20 ng/mL IL-2 for addition to myotube cultures. Different effector to target (E:T) ratios of 0.5:1, 1:1, 2.5:1, and 5:1 of TALL-104 cells were added based on the initial seeding density of myogenic cells for 4, 24, or 48 h. For ELISA (enzyme linked immunosorbent assay) and immunofluorescent experiments analysing TDP-43 and p62, 1:1 E:T ratio for 24 h was used.

### Cytotoxicity assay

Cytotoxicity was measured using CytoTox 96® non-radioactive cytotoxicity assay (Promega, UK G1780) following manufacturer’s instructions. Cytotoxicity was measured in medium samples from the cultured myotubes after TALL-104 coculture or IL-2 control. Substrate solution was added to samples before addition of stop solution, and optical density read at 492 nm using a Multiskan™ FC plate reader. Results were normalised to a medium-only control and a positive cytotoxicity control, where cells were incubated in lysis solution for 45 min before commencing the assay. Cytotoxicity was expressed as a percentage of the positive lysis control.

### Immunofluorescent microscopy

Skeletal muscle-derived cells were seeded on Gibco Geltrex (A1569601)-coated 13 mm round coverslips in 24 well plates. The cells were allowed to proliferate for 48 h before differentiation for 5 days before coculture with TALL-104 cells for 24 h. Cells were washed and fixed with 4% formaldehyde solution (Fisher Scientific, UK 12777847) for 10 min at room temperature. Cells were permeabilised with 0.1% v/v triton X100 in PBS for 15 min before blocking with 5% goat serum in PBS for 30 min to 1 h. Primary antibodies TDP-43 1:150 (10782–2-AP) and p62 1:100 (66184–1-Ig, both from Proteintech, UK) diluted in blocking buffer were added by inverting the coverslip onto a 30 µL drop of antibody solution on Parafilm®, and incubating in a humidified chamber overnight at 4 °C. Coverslips were incubated in appropriate Alexa Fluor™-conjugated secondary antibodies (Invitrogen,UK Alexa Fluor™ goat anti-rabbit 488 A-11008 or goat anti mouse 546 A-11030) for 1 h at room temperature. Cells were washed and HCS CellMask™ deep red (H32721) was added at 1:5000 in PBS for 30 min to visualise all cell contents. Afterwards, DAPI (4′,6-diamidino-2-phenylindole, R37606) at 5 μg/mL in PBS was added for 5 min. After washing, cells were mounted onto glass slides with DAKO fluorescence mounting medium (Agilent, USA).

### TDP-43 localisation

To analyse TDP-43 localisation, images were captured using an Olympus IX81-ZDC inverted widefield fluorescent microscope at 10× magnification. Four images were analysed per condition per myogenic donor. DAPI and HCS CellMask™ staining was used to observe subcellular compartments of the nucleus and sarcoplasm respectively. Based on the observed staining, TDP-43 was classified as being within the nucleus, sarcoplasm, nucleus and sarcoplasm, or neither (not expressed). Localisation was quantified for both single nucleated and multinucleated cells. Localisation was expressed as a percentage of total number of cells observed with HCS CellMask™ for each localisation compartment.

### TD-43 and p62 aggregation analysis

For p62 and TDP-43 aggregation analysis, cells were imaged using a Zeiss LSM 800 confocal microscope. Eight z-stacks each composed of two images approximately 0.9 µm apart from one experiment were captured. Images were analysed using NIH Image J (USA). Obtained images were split into individual colour channels and converted to 8-bit. p62 and HCS CellMask™ channels were manually thresholded.

p62 particle frequency and size was determined in the p62 channel using NIH Image J using the “analyse particles” function with pixel size over 3. p62 frequency was the total number of particles obtained per image with the “analyse particles” function and p62 size was the average particle size per image. In the thresholded cell mask channel, percentage area of image coverage was obtained. p62 particle frequencies were normalised to percentage area coverage per image using HCS CellMask™ staining, to account for differences in myotube size.

The same images for p62 analysis were used for TDP-43 sarcoplasmic aggregate analysis. Sarcoplasmic aggregates were identified manually as punctate areas of staining. Using Image J, freehand regions were drawn around aggregates to measure their area in µm^2^. TDP-43 aggregate frequencies were normalised to percentage area coverage per image of cell mask staining using the cell mask coverage. TDP-43 colocalisation with p62 was assessed for each identified TDP-43 aggregate using thresholded p62 and TDP-43 channels. Any area of overlap between TDP-43 and p62 objects over two pixels was considered colocalised. Percentage colocalisation is the percentage of TDP-43 aggregates that colocalised with p62 puncta for all aggregates observed for one donor under one condition. p62 puncta and TDP-43 aggregate analysis was conducted blinded.

### ELISA

DuoSet® Enzyme-linked immunosorbent assays (ELISA) were purchased from R&D systems, UK. Human IFN gamma (DY285B), and human TNF alpha (DY210) were used. ELISA were performed following manufacturer’s instructions. Nunc MaxiSorp™ immunoplates were coated with recommended dilutions of capture antibody, blocked with 0.2 µm filtered 1% w/v BSA (Sigma, UK, A7030) (reagent diluent) for 1 h, before incubation of samples and standards diluted in reagent diluent for 2 h. Recommended concentrations of detection antibody diluted in reagent diluent were added for 2 h in sealed plates. Streptavidin horse radish peroxidase (HRP) diluted in reagent diluent was incubated for 20 min before washing and addition of substrate solution (1:1 mix hydrogen peroxide and tetramethylbenzidine, R&D Systems, UK) for 20 min. Colour development was halted with 2N sulphuric acid. Optical density was determined using a Multiskan™ FC plate reader by subtracting readings at 540 nm from 450 nm to account for any imperfections in the plate.

### Statistics

Statistical analysis and graph plotting was performed with GraphPad Prism 7. Normal distribution of data was tested with Shapiro Wilk test before choosing appropriate statistical tests. All statistical tests performed were two-tailed. Data are displayed as mean ± standard error of mean for normally distributed data or median ± interquartile range for non-normally distributed data.

## Results

### TALL-104 coculture was cytotoxic to myotubes

To examine if direct coculture with TALL-104 cells was cytotoxic to myotubes, lactate dehydrogenase release was measured from cocultures at different E:T ratios after 4, 24, and 48 h. The E:T ratios used were 0.5:1, 1:1, 2.5:1, and 5:1. IL-2-containing medium was used as a control. IL-2 is a cytokine whose main roles are within the immune system^34^, therefore it was not expected that the inclusion of this cytokine in controls would have any detrimental effect on myogenic cells, and IL-2-containing medium showed low myotube cytotoxicity. These myogenic cultures show limited continuous differentiation after day 4 (supplementary Fig. S1), therefore IL-2 is not expected to interfere with differentiation.

**Figure 1:**
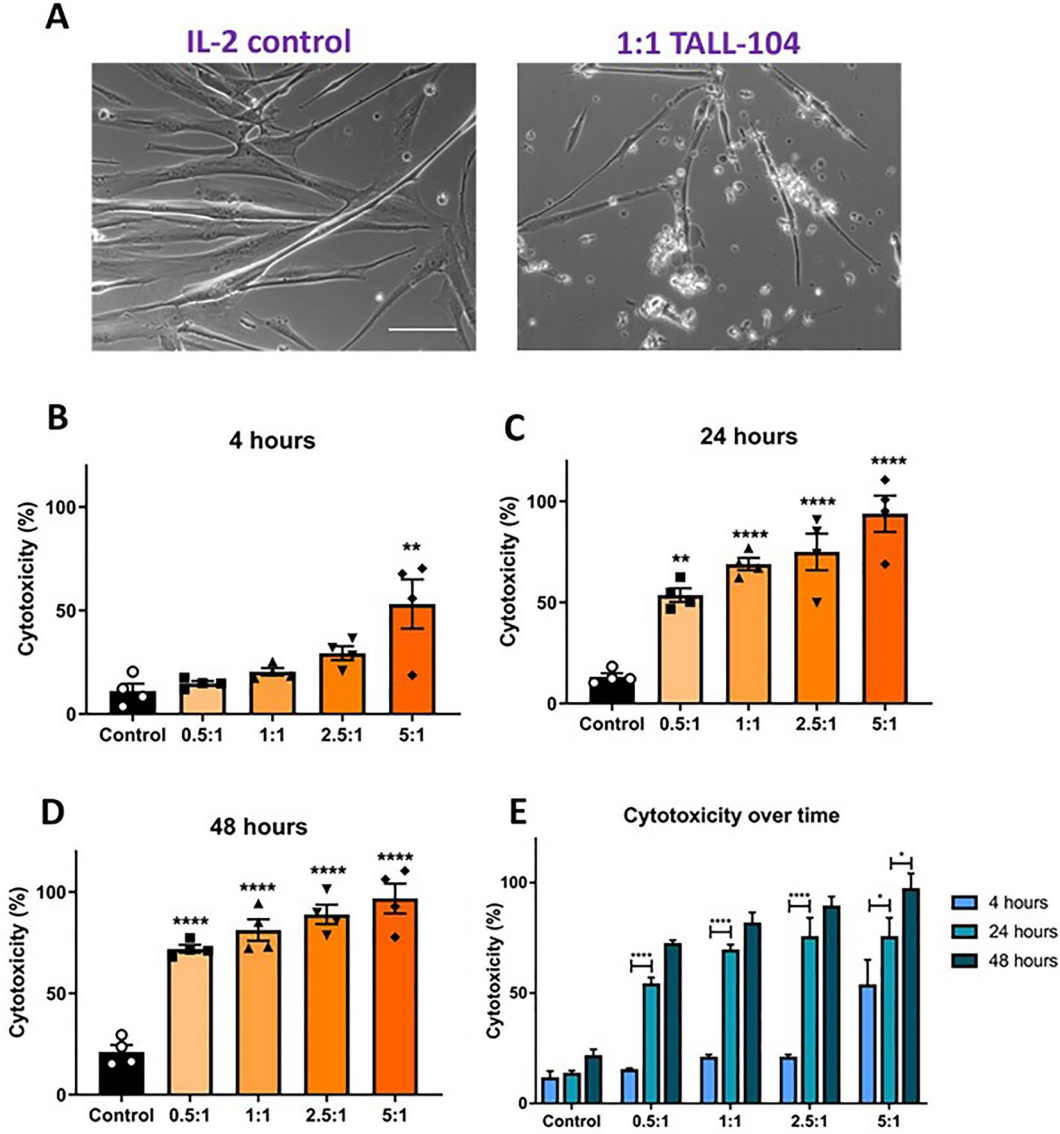
TALL-104 coculture was cytotoxic to myotubes. (**A**) Phase contrast images of myotubes with IL-2 control or 1:1 TALL-104 effector to target (E:T) ratio after 24 h, with TALL-104 visible as bright, irregularly shaped cells. Fewer myotubes were observed with coculture, and remaining myotubes appeared thinner. Scale bar = 100 µm. (**B**–**D**) Cytotoxicity of different E:T ratios compared to IL-2 control. (**B**) At 4 h of coculture, there was higher cytotoxicity in the 5:1 E:T condition compared to control (*p* = 0.0005), but not for any other E:T ratio. (**C**) At 24 h there was higher cytotoxicity for 0.5:1, 1:1, 2.5:1, and 5:1 compared to control (*p* = 0.001 0.5:1, *p* = 0.0001 all other comparisons). (**D**) At 48 h, there was higher cytotoxicity for 0.5:1, 1:1, 2.5:1, and 5:1 compared to control (*p* = 0.0001 for all comparisons). (**E**) For each E:T ratio, comparison of cytotoxicity between timepoints. There was no difference in cytotoxicity over time in the control group. Cytotoxicity was higher at 24 h than 4 h for 0.5:1, 1:1, 2.5:1 (*p* < 0.0001), and 5:1 (*p* = 0.0223). Cytotoxicity was also higher at 48 h than 24 h for 5:1 (*p* = 0.0226). For all E:T ratios 48 h had higher cytotoxicity than 4 h. (**B**–**D**) One-way ANOVA with Dunnett’s multiple comparisons. (**E**) Two-way ANOVA with Tukey’s multiple comparisons. n = 4 myogenic donors.

In cocultures, large areas of floating debris surrounded by TALL-104 cells were observed. Myotubes that remained attached to the surface appeared thinner than those in the control condition (Fig. 1A). At 4 h, only the highest E:T ratio of 5:1 showed a significant increase in cytotoxicity compared to myotubes cultured with IL-2 control (Fig. 1B). By 24 h, all E:T ratios showed increased cytotoxicity of myotubes compared to no TALL-104 IL-2 control, which was also true for 48 h co-culture (Fig. 1C,D). At 24 h, the mean percentage cytotoxicity was 13.23 ± 1.73% in the control, 53.66 ± 3.4% for 0.5:1, 68.97 ± 3.04% 1:1, 75.02 ± 9.07% 2.5:1, and 93.91 ± 8.92% 5:1. When comparing cytotoxicity over time, all E:T ratios showed higher cytotoxicity at 24 h compared to 4 h, whereas only 5:1 ratio showed increased cytotoxicity from 24 to 48 h, suggesting most cytotoxic effects occur between 4 and 24 h (Fig. 1E).

### Coculturing TALL-104 with myotubes lead to increased IFNγ and TNFα secretion from TALL-104 at higher E:T ratio

**Figure 2:**
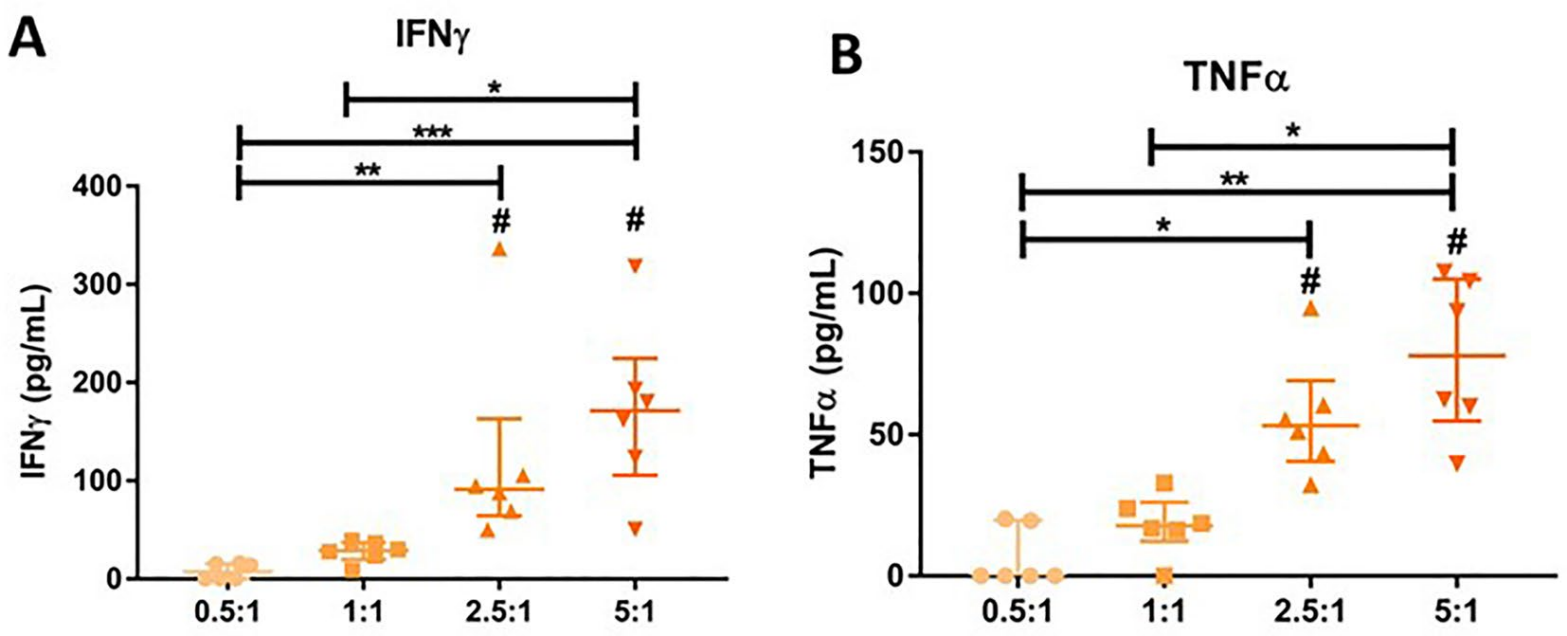
IFNγ and TNFα secretion from TALL-104 cells co-cultured with myotubes. Different effector (TALL-104):target (myotube) ratios were used. (**A**) IFNγ secretion was higher when cocultured with myotubes than with TALL-104 alone for 2.5:1 and 5:1 ratios (#, *p* = 0.0196 2.5:1, *p* = 0.0009 5:1. Two-way ANOVA with Sidak multiple comparisons). No IFNγ was detected from any ratio of TALL-104 cultured alone. IFNγ secretion was higher in 5:1 vs 0.5:1 (*p* = 0.0006), 5:1 vs 1:1 (*p* = 0.0475), and 2.5:1 vs 0.5:1 (*p* = 0.0075). Kruskal–Wallis test with Dunn’s multiple comparisons. When considering the median IFNγ values, the increase in IFNγ with increasing E:T ratio was mostly linear (r^2^ = 0.9245). (**B**) TNFα secretion was higher when cocultured with myotubes than with TALL-104 alone for 2.5:1 and 5:1 ratios (#, *p* = 0.0001 2.5:1, *p* < 0.0001 5:1. Two-way ANOVA with Sidak multiple comparisons). TNFα secretion was higher for 5:1 than 0.5:1 (*p* = 0.0016), 5:1 vs 1:1 (*p* = 0.0189), and 2.5:1 vs 0.5:1 *p* = 0.0246). Kruskal–Wallis test with Dunn’s multiple comparisons. When considering the median TNFα values, the increase in TNFα with increasing E:T ratio was mostly linear (r^2^ = 0.9335). n = 6 myogenic donors. Median ± IQR.

To examine whether coculturing TALL-104 cells with myotubes affected activation of TALL-104 cells, ELISA was used to measure secreted levels of IFNγ and TNFα in the culture medium. IFNγ and TNFα levels were measured after 24 h TALL-104-myotube coculture. This was compared to TALL-104 cells cultured in myotube medium without myotubes as a negative control, where IFNγ and TNFα were not detected above 1 pg/mL. There was higher IFNγ and TNFα secretion at 2.5:1 and 5:1 compared to monocultured TALL-104 (Fig. 2). Furthermore, there was higher IFNγ and TNFα secretion at 5:1 compared to either 1:1 and 0.5:1 E:T ratio, and higher secretion of both cytokines at 2.5:1 than 0.5:1. The median IFNγ release was 7 ± 16, 29 ± 18, 91 ± 99, and 171 ± 119 pg/mL for 0.5:1, 1:1, 2.5:1, and 5:1 respectively. The median TNFα release was 0 ± 20, 18 ± 14, 53 ± 28, and 78 ± 50 pg/mL for 0.5:1, 1:1, 2.5:1, 5:1 respectively. There was a linear increase of both IFNγ (r^2^ = 0.9245) and TNFα (r^2^ = 0.9335) secretion with increasing E:T ratio when considering only the median concentration value for each condition, showing increased cytokine secretion was due to higher TALL-104 cell number.

### TALL-104 cells attached to and invaded myotubes

**Figure 3:**
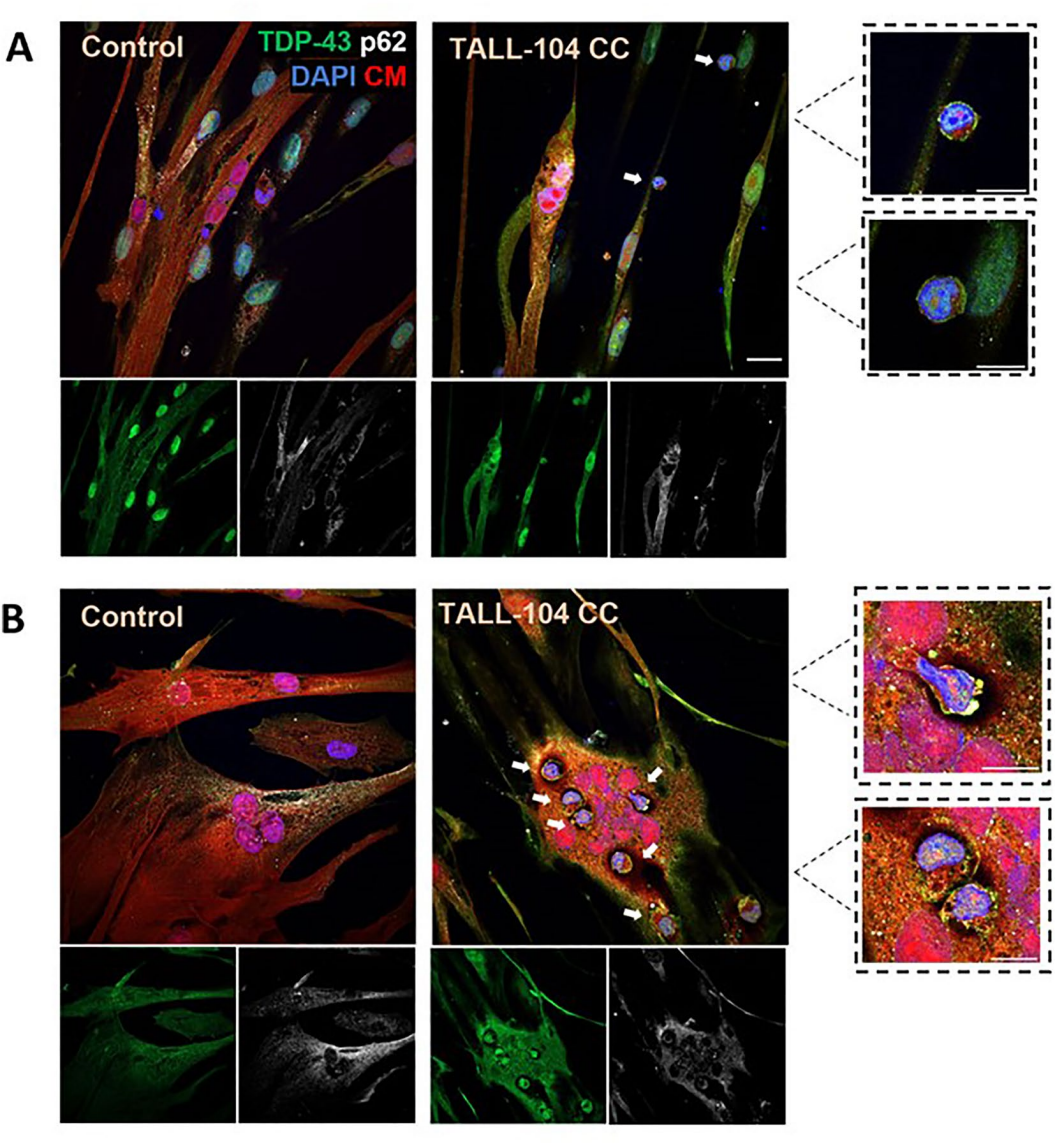
Attachment and invasion of myotubes by TALL-104 cells. Images captured at 1:1 E:T ratio after 24 h. TALL-104 were visible as small cells with high nuclear to cytoplasmic ratio**.** (**A**) TALL-104 cells attached to myotubes. (**B**) TALL-104 cells invading the sarcoplasm of myotubes, some showing morphological changes (top inset). Scale bar = 20 µm, inset scale bar = 10 µm. White arrows indicate TALL-104 cells. CM—cell mask, CC—coculture.

TDP-43 and p62 immunofluorescent staining was conducted in myotube TALL-104 cocultures. In some immunofluorescent images of cocultures, small rounded cells with a high nuclear to cytoplasm ratio were observed which were likely TALL-104 cells. TALL-104 cells were observed attaching to myotubes (Fig. 3A). They were also seen in the sarcoplasm of myotubes, with a halo of darkness surrounding them, which may show localised myotube destruction (Fig. 3B). Furthermore, TDP-43 and p62 were visible in TALL-104 cells. TDP-43 was observed in the nucleus of TALL-104 cells, as well as strongly staining the edges of the cells. p62 was less pronounced but was also localised towards the periphery of the cell cytoplasm.

### TALL-104 coculture did not affect p62 and TDP-43 aggregation, but TDP-43 aggregates were less likely with coculture

**Figure 4:**
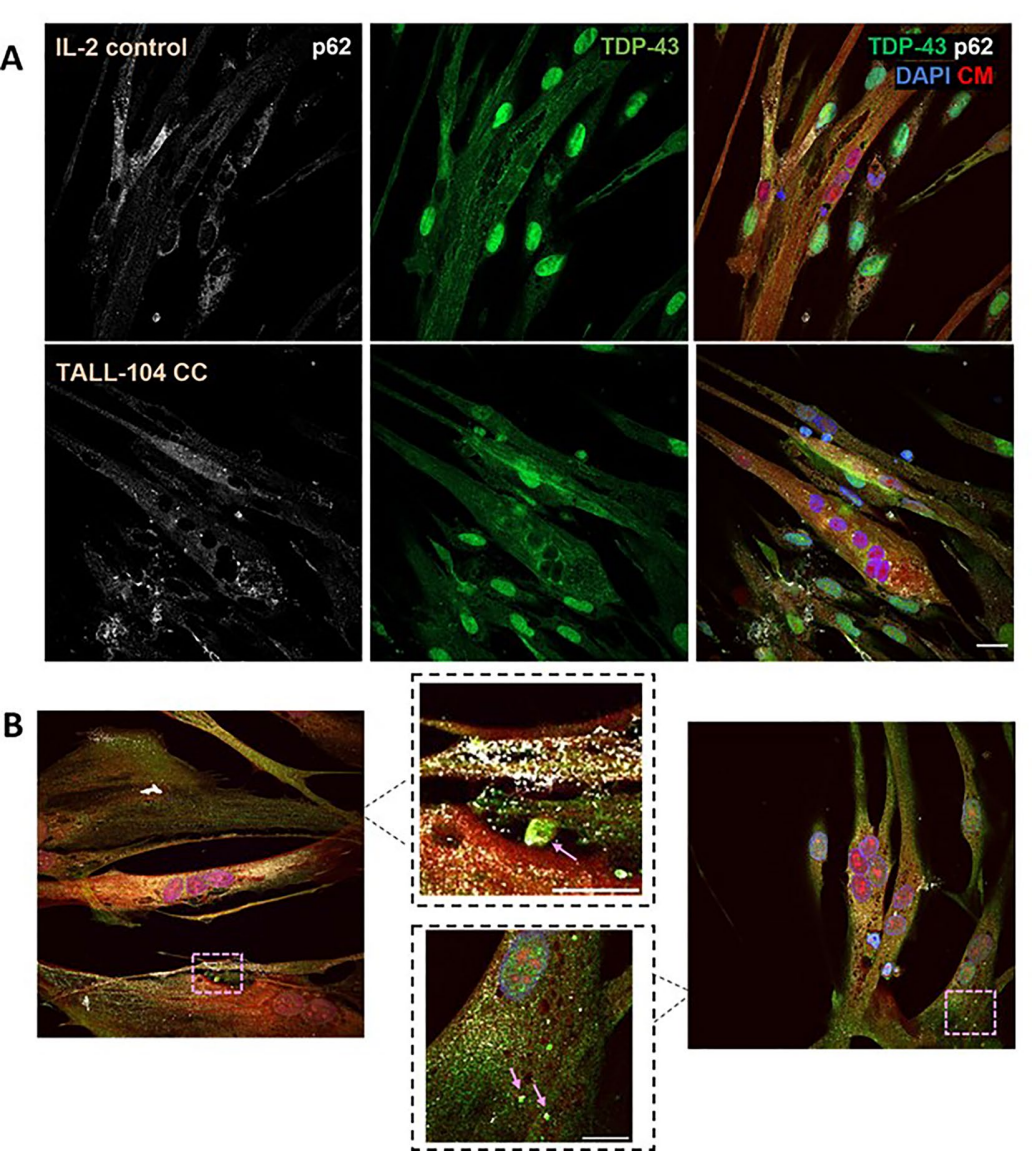
p62 and TDP-43 in myotubes treated with IL-2 control or TALL-104 coculture. Representative images of TDP-43 and p62 in IL-2 control or TALL-104 coculture (CC) treated myotubes. CM- cell mask. (**B**) TDP-43 and p62 in TALL-104 cocultured myotubes from two different donors. Insets show TDP-43 aggregates with partial p62 colocalisation. Scale bar = 20 µm, inset scale bar = 10 µm.

**Figure 5:**
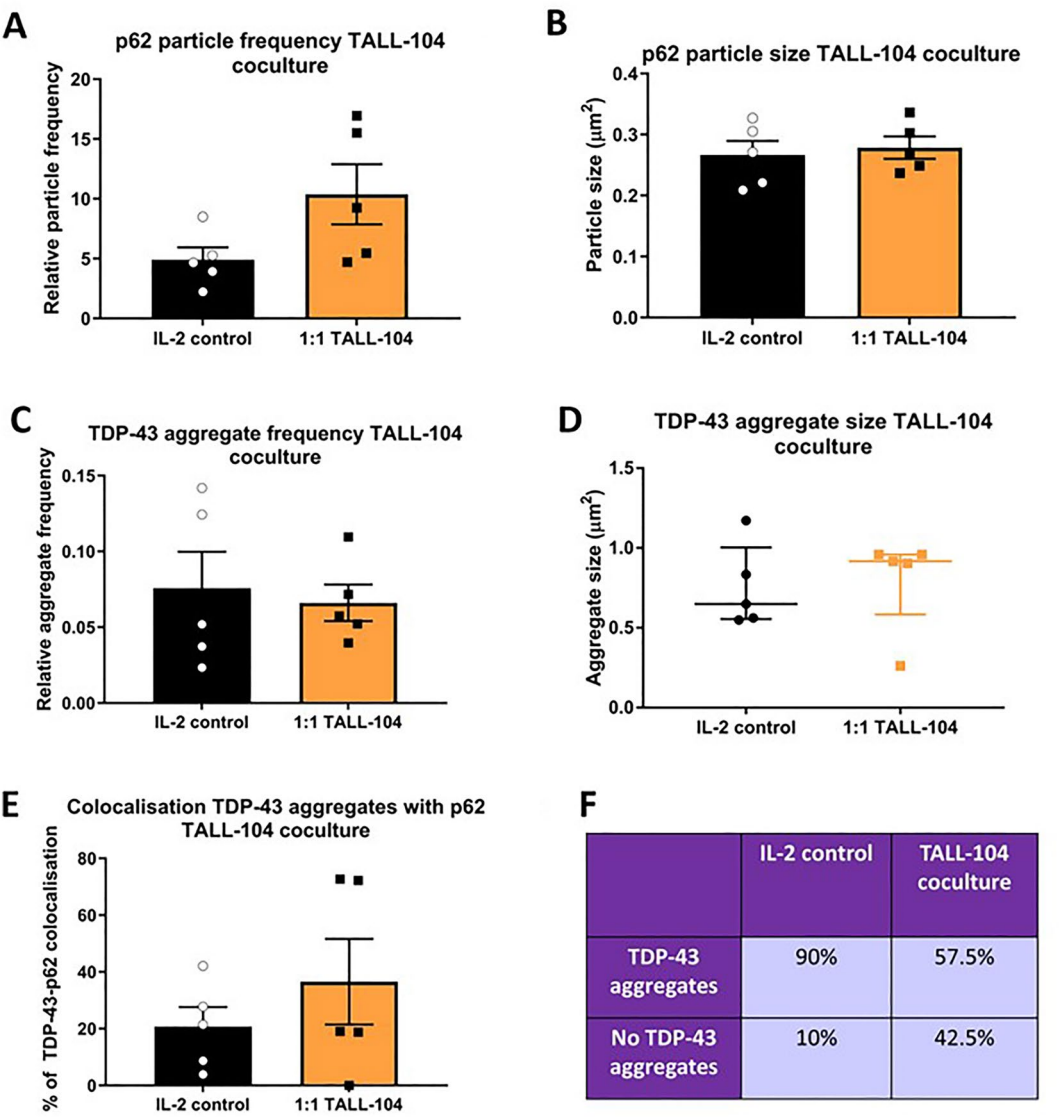
Coculturing TALL-104 cells with myotubes did not affect p62 or TDP-43 aggregate size or frequency. (**A**) There was no difference in p62 puncta frequency between IL-2 control and TALL-104 coculture at 1:1 effector to target ratio (*p* = 0.0796). (**B**) There was no difference in p62 puncta size between IL-2 control and TALL-104 coculture (*p* = 0.697). Student’s T-test. (**C**) There was no difference between IL-2 control and TALL-104 co-culture for TDP-43 aggregate frequency relative to cell area (*p* = 0.728), (**D**) aggregate size (*p* = 0.548), (**E**) or colocalisation with p62 puncta (*p* = 0.369). (**F**) There was a higher frequency of images containing TDP-43 aggregates in the IL-2 control group compared to TALL-104 coculture (Fisher’s exact test *p* = 0.0018, eight images (with two z-stacks) for each of 5 donors). (**A**–**E**) Student’s T-test or Mann–Whitney U test. n = 5 myogenic donors.

To examine if direct coculture with cytotoxic immune cells caused p62 aggregation in myotubes, immunofluorescent microscopy was used to quantify size and frequency of p62 puncta. 24 h coculture with 1:1 E:T ratio of TALL-104 cells was used. Figure 4A shows p62 in myotubes. p62 was mostly located in the sarcoplasm with variable sized puncta and some areas of diffuse staining, occasional p62 puncta in the nucleus could also be observed. p62 puncta frequency relative to cell area was not affected by TALL-104 coculture compared to IL-2 control (Fig. 5A). There was also no difference in p62 puncta size between IL-2 control and TALL-104 coculture (Fig. 5B) with mean p62 puncta size of 0.267 ± 0.023 µm^2^ in the IL-2 control group.

Immunofluorescent microscopy was also used to assess TDP-43 sarcoplasmic aggregation with TALL-104 exposure. Figure 4A,B shows TDP-43 staining in myotubes. TDP-43 was diffusely located throughout the cytoplasm. In untreated control myotubes, TDP-43 aggregates and p62 puncta were also observed (supplementary Fig. S2, Table S1). Some myotubes showed nuclear TDP-43 staining whereas others showed an absence of TDP-43 in the nucleus. The frequency of sarcoplasmic TDP-43 aggregates relative to myotube area was not different between myotubes cultured with IL-2 control or TALL-104 cells (Fig. 5C). There was also no difference in TDP-43 aggregate size (Fig. 5D) or co-localisation with p62 puncta (Fig. 5E) between control and TALL-104 coculture. The median TDP-43 aggregate size in the IL-2 control group was 0.65 ± 0.45 µm^2^. TDP-43 aggregates were not observed in all images. There was a lower frequency of images containing any TDP-43 aggregates in the TALL-104 coculture group than the IL-2 control group (Fisher’s exact test *p* = 0.0018 Fig. 5F). There was no difference in the median number of cells imaged in each condition (*p* = 0.651, 3 ± 2.3 vs. 2.9 ± 3.3 for IL-2 control and TALL-104 coculture respectively, median ± IQR).

### TDP-43 subcellular localisation was affected by TALL-104 coculture

**Figure 6:**
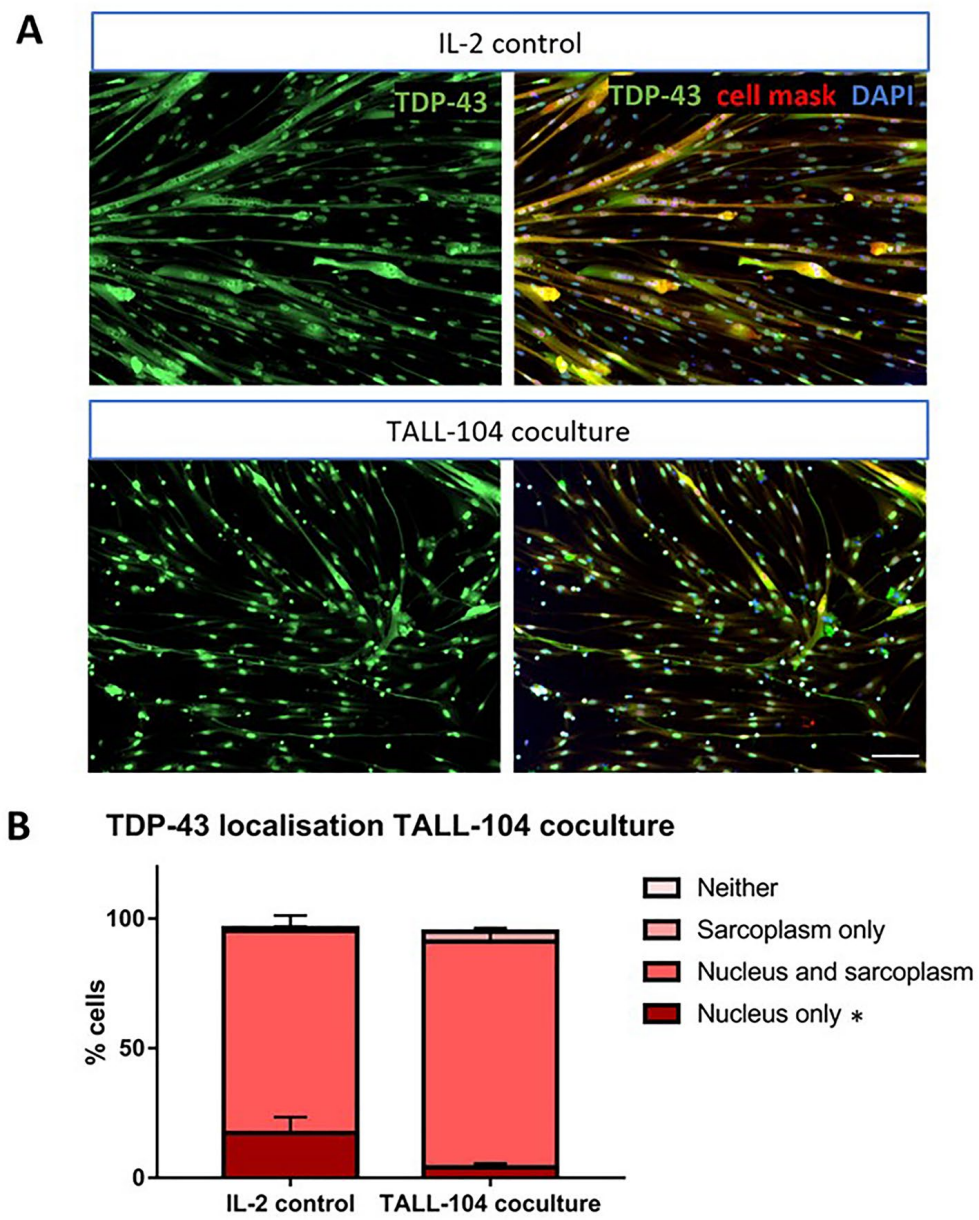
TDP-43 subcellular localisation was affected by TALL-104 coculture. (**A**) Representative images of TDP-43 localisation with IL-2 control and TALL-104 coculture. Scale bar = 100 µm. (**B**) There was a difference in the distribution of TDP-43 between the subcellular compartments between the two treatment conditions (two-way ANOVA interaction factor *p* = 0.02). There was a decrease in nuclear only expression of TDP-43 with TALL-104 coculture compared to IL-2 control (*p* = 0.0401), but there was no difference for the nuclei and sarcoplasm (*p* = 0.241), sarcoplasm only (*p* = 0.974), or neither (*p* > 0.999) groups. Two-way ANOVA with Sidak’s multiple comparisons. n = 5 myogenic donors.

The localisation of TDP-43 within myogenic cells was assessed when cultured with 1:1 E:T ratio of TALL-104 for 24 h compared to skeletal muscle derived cells treated with IL-2-containing media for the same length of time. There was a significant difference in the interaction between TDP-43 localisation and treatment condition, showing TDP-43 localisation was altered with TALL-104 coculture (two-way ANOVA) (Fig. 6). The only subcellular localisation with a difference between IL-2 control and TALL-104 coculture with Tukey’s post-hoc testing was “nucleus only”, showing fewer cells with TDP-43 only in the nucleus in TALL-104 coculture compared to IL-2 control. In immunofluorescent images of the TALL-104 coculture group there appeared more cells with weak TDP-43 sarcoplasmic (and nuclear) expression. There was also a significant difference in the localisation of TDP-43 between the subcellular compartments (two-way ANOVA *p* < 0.0001). The localisation with the largest percentage of TDP-43 regardless of treatment condition was nucleus and sarcoplasm with mean 83 ± 3.71% (Tukey’s post-hoc test).

## Discussion

This study aimed to investigate the effects of cell-mediated cytotoxicity on TDP-43 and p62 degenerative features in skeletal muscle. Other proteins are also implicated in sIBM, most notably amyloid-β^35–37^. However, the importance of amyloid-β in sIBM has been called into question^38^. The presence of amyloid β aggregates in sIBM myofibres may be low, with some studies showing as low as between 0^39^ and 0.4%^40^ of myofibres containing amyloid deposits. On the other hand, amyloidβ was found over-represented in rimmed vacuoles compared to other areas of the sarcoplasm^15^. It was decided not to focus on this protein here. Whilst TDP-43 and p62 are suggested as specific sIBM markers, aggregates of these proteins have been observed in other muscular diseases. TDP-43 aggregation is seen in oculopharyngeal muscular dystrophy, desminopathy, myotilinopathy, and hereditary IBM^41,42^, however, these diseases are not immune-mediated. With no distinctive TDP-43 aggregate pattern in sIBM compared to other diseases^42^, the combined presence of TDP-43 and CD8 + T cells may serve as selective markers of sIBM. p62 is identified in other inflammatory myopathies, particularly immune-mediated necrotising myositis (IMNM) but undetectable in normal muscle^43,44^.

This study builds upon previous work examining cytotoxic immune cell interactions with skeletal muscle cells. Coculture of antigen-specific CD8 + T cells and transgenic antigen presenting myotubes was used to model polymyositis. These myotubes died with increasing lysis at increasing densities of CTLs and the CD8 T cells infiltrated into the myotubes^45^, similar to what was observed here. The effect of coculturing isolated sIBM CD8 + T cells with autologous myotubes has been tested, showing 1 out of 4 sIBM patient’s CTLs showed cytotoxicity against autologous myotubes^46^. This suggests some sIBM patient CTLs may be primed against skeletal muscle antigens.

Previous in vitro models of sIBM have been created. IL-1β and IFNγ treatment of healthy human myotubes was used to investigate the effects of these cytokines on a range of degenerative characteristics altered in sIBM^47–49^. In rat myotubes, treatment with IL-1β triggered TDP-43 mislocalisation to the sarcoplasm, without triggering inclusion body-like protein aggregates^50^. These results show inflammation can precede non-inflammatory sIBM features in myotubes. However, sIBM-derived myotubes cultured under electrical stimulation showed TDP-43 and p62 aggregation^51^, highlighting a potential muscle-intrinsic mechanism of aggregation in sIBM. The effects of cell-mediated cytotoxic immunity on TDP-43 and p62 in myotubes has not previously been investigated. The work presented here contributes to understanding the interplay of inflammation on degenerative sIBM-like features.

Here, TALL-104 cells were used as a proxy for CD8 T cells due to their continued growth in culture and easy availability. TALL-104 is a lymphoblastic leukaemic cells line expressing CD8, CD3, and T cell receptor proteins as well as natural killer (NK) cell markers including CD56 and CD161^32,33^. Blocking NK cell receptors NKp46, NKG2D, and 2B4 prevented TALL-104-mediated lysis, therefore cytotoxicity is mediated through NK cell receptors, independent of MHC recognition^32^. Instead, they resemble a mixed lineage sharing features of NK and CD8 T cells^52–56^.

It was expected TALL-104 coculture would not be cytotoxic as myotubes should lack epitopes that trigger cytotoxicity. Furthermore, TALL-104 spare normal cells whilst targeting tumourigenic cells^57–60^. NK cells can target autologous skeletal muscle cultures from healthy patients, whilst autologous myotubes were not targeted or killed by CD8 cytotoxic T cells^61^. This suggests culturing myogenic cells may reveal epitopes capable of stimulating NK cells that are usually hidden or absent *in viv*o. The lack of reactivity of TALL-104 cells to normal brain cells^57^ indicates cytotoxicity against myotubes is unlikely to be due to HLA-mismatching or anti-self recognition.

The results presented here suggest exposure to cytotoxic immune cells does not precede TDP-43 and p62 sarcoplasmic aggregation. The decreased likelihood of images containing TDP-43 aggregates may reflect a change in homeostatic functions of TDP-43, such as RNA processing in myo-granules^62^ where TDP-43 forms puncta with muscle-specific mRNAs. In cells undergoing apoptosis there is a rapid degradation of mRNA^63^, therefore TDP-43’s homeostatic roles could cease with TALL-104 coculture. However, selection bias where only cells with a higher propensity to survive TALL-104 cytotoxicity may have influenced these results. Whilst a decrease in nuclear TDP-43 was observed with TALL-104 coculture, the localisation did not shift to the sarcoplasm only, showing a “mislocalisation” of TDP-43 was not recapitulated. However, this suggests cytotoxic inflammatory cell exposure can contribute to TDP-43 subcellular shifts, which could contribute to the overall phenotype of TDP-43 mislocalisation as seen in sIBM.

There are several limitations to this study. Firstly, TALL-104 cells mediate cytotoxicity through MHC-independent mechanisms, as well as being a neoplastic cell line. sIBM CD8 + T cells are highly differentiated, therefore the TALL-104 cell line is unlikely to share similar activation features to native sIBM CTLs. TALL-104 were identified based on size and high nuclear to cytoplasmic ratio, but co-staining with CD8 or other T cell markers would have been preferrable. The inflammatory state of the myotubes themselves was not tested, which could have been investigated by examining major histocompatibility complex I and other inflammatory markers on myotubes. To further characterise the cytotoxic reactivity of TALL-104 to myotubes, granzyme and perforin analysis could be conducted. Finally, in comparison to the slowly progressive nature of sIBM, the cytotoxicity of TALL-104 cells here was acute, limiting the relevance of these experiments.

Overall, the exploratory results in this study show exposure to cytotoxic immune cells did not trigger TDP-43 or p62 sarcoplasmic aggregation as seen in sIBM, suggesting in vitro aggregation of these proteins were not preceded by inflammatory cytotoxicity. Conversely, TALL-104 cells triggered a shift in TDP-43 subcellular localisation, showing cytotoxic cells may contribute to the sIBM-like characteristic of TDP-43 mislocalisation to the sarcoplasm. This study helps towards understanding whether inflammation or protein aggregation arises first in sIBM.

### Supplementary Information

Supplementary Information.

## Data Availability

All analysed data are included in this published article (and its Supplementary Information files). Raw data available upon reasonable request from the corresponding author.
