# Supplementary material for: Cytotoxic immune cells do not affect TDP-43 and p62 sarcoplasmic aggregation but influence TDP-43 localisation
Source: Sci Rep. 2023 Sep 23;13:15935. doi: 10.1038/s41598-023-42824-5 (PMC10517962; doi:10.1038/s41598-023-42824-5)
Supplement: Supplementary file 1 — Supplementary Information. [file 41598_2023_42824_MOESM1_ESM.docx]

**Cytotoxic Immune Cells Do Not Affect TDP-43 and p62 Sarcoplasmic Aggregation But Influence TDP-43 Localisation**

Bryony McCord, Richard M. Day

Centre for Precision Healthcare, UCL Division of Medicine, University College London, London, WC1E 6JF, United Kingdom


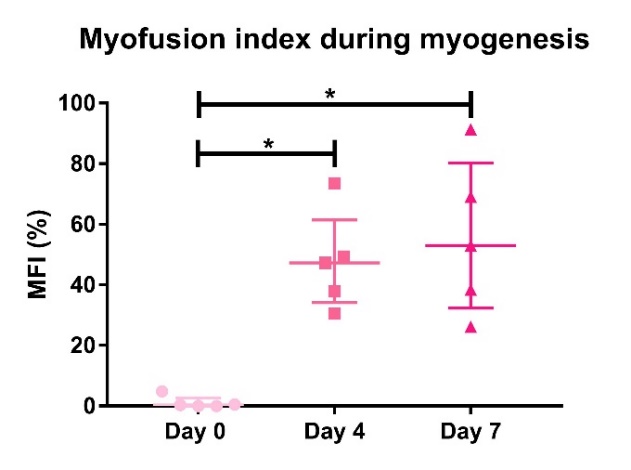

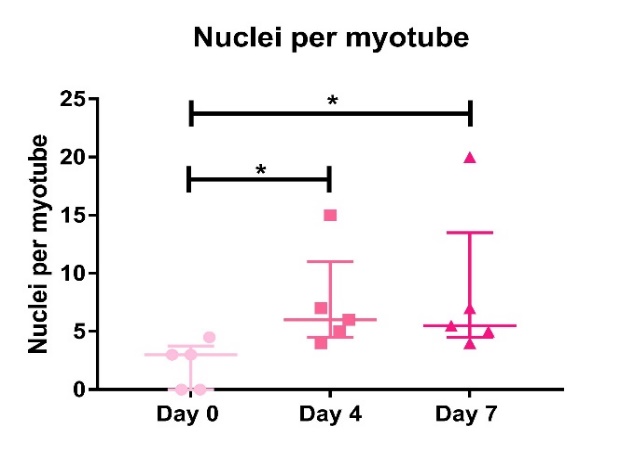
Figure S1. Differentiation over time. Myogenic cells were assessed for nuclei per myotube (A) and myofusion index (MFI) (B) on day 0 (proliferation), day 4, and day 7 of differentiation. (A) number of nuclei per myotube with a minimum of three nuclei. There was a difference in number of nuclei between day 0 and 4 (p = 0.0428) and day 0 and 7 (p = 0.0428), but not between day 4 and day 7. (B) MFI expressed as the percentage of nuclei in a myotube (minimum three nuclei) compared to total nuclei count. There was a difference between day 0 and 4 (pp = 0.0397), and day 0 and 7 (p = 0.0139), but not day 4 and 7. Kurskal Wallis with Dunn’s multiple comparisons, n = 5 myogenic donors.

**B**

**A**


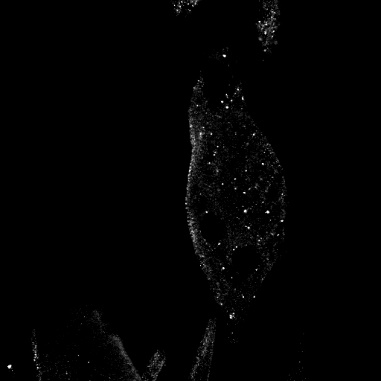

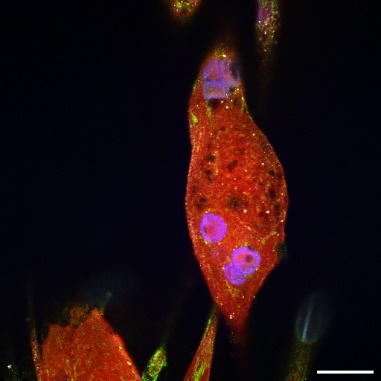

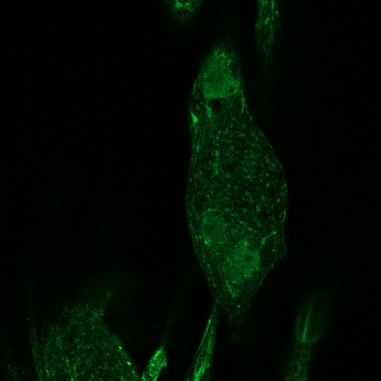


**p62**

**TDP-43**

**p62 TDP-43
DAPI Cell mask**

Figure S2. Untreated control myotubes. p62 puncta and TDP-43 aggregates were present in untreated myotubes after 9 days differentiation. Scale bar = 20 µm.

| **Measurement** | **Untreated control average** | **IL-2 control average** | **Statistical comparison** |
| --- | --- | --- | --- |
| **p62 relative puncta frequency** | 6.16 ± 1.42 | 4.91 ± 2.31 | Student’s T test p = 0.511 |
| **p62 puncta size** | 0.536 ± 0.11 µm^2^ | 0.279 ± 0.018 µm^2^ | Student’s T test p = 0.0662 |
| **TDP-43 relative aggregate frequency** | 0.0986 ± 0.0333 | 0.0758 ± 0.024 | Student’s T test p = 0.607 |
| **TDP-43 aggregate size** | 0.669 ± 0.722 µm^2^ | 0.650 ± 0.447 µm^2^ | Mann-Whitney U test p = 0.931 |
| **Percentage images containing any TDP-43 aggregate** | 62.5 % | 90 % | Fisher’s Exact test p = 0.0116 |
| **TDP-43 localisation** | Nucleus: 23.9 % Nucleus+sarcoplasm: 41.4 %  Sarcoplasm: 24.3 %  Neither: 10.4 % | Nucleus: 17.3 % Nucleus+sarcoplasm: 78 %  Sarcoplasm: 1.1 %  Neither: 0 % | Two-way ANOVA (treatment condition comparison)  p = 0.724 |

Table S1. Statistical comparisons of TDP-43 and p62 features between untreated control condition (n = 6 except TDP-43 localisation n = 5) and IL-2 control (n = 5) measured in different experiments. In the untreated control condition, myotubes were differentiated for 9 days compared to 5 + 1 day IL-2 treatment in the IL-2 control. For TDP-43 and p62 size and frequency, three myogenic donors were the same in each experimental group. The reported averages are mean ± standard error of mean except for TDP-43 aggregate size which is median ± interquartile range.
